# Association between Prediagnostic Allergy-Related Serum Cytokines and Glioma

**DOI:** 10.1371/journal.pone.0137503

**Published:** 2015-09-09

**Authors:** Judith Schwartzbaum, Michal Seweryn, Christopher Holloman, Randall Harris, Samuel K. Handelman, Grzegorz A. Rempala, Ruo-Pan Huang, Brett Burkholder, Adam Brandemihl, Henrik Kallberg, Tom Borge Johannesen, Anders Ahlbom, Maria Feychting, Tom K. Grimsrud

**Affiliations:** 1 Division of Epidemiology, College of Public Health, Ohio State University, Columbus, Ohio, United States of America; 2 Comprehensive Cancer Center, Ohio State University, Columbus, Ohio, United States of America; 3 Division of Biostatistics, College of Public Health, Ohio State University, Columbus, Ohio, United States of America; 4 Mathematical Biosciences Institute, Columbus, Ohio, United States of America; 5 Department of Mathematics, University of Lodz, Lodz, Poland; 6 Department of Statistics, Ohio State University, Columbus, Ohio, United States of America; 7 Center for Pharmacogenetics, Wexner Medical Center, Ohio State University, Columbus, Ohio, United States of America; 8 RayBiotech, Inc., Norcross, Georgia, United States of America; 9 RayBiotech, Inc. Guangzhou, China; 10 Buckeye Psychiatry, Columbus, Ohio, United States of America; 11 Institute of Environmental Medicine, Karolinska Institutet, Stockholm, Sweden; 12 Department of Registration, Cancer Registry of Norway, Oslo, Norway; 13 Department of Research, Cancer Registry of Norway, Oslo, Norway; University Hospital of Heidelberg, GERMANY

## Abstract

Allergy is inversely related to glioma risk. To determine whether prediagnostic allergy-related serum proteins are associated with glioma, we conducted a nested case-control study of seven cytokines (IL4, IL13, IL5, IL6, IL10, IFNG, TGFB2), two soluble cytokine receptors (sIL4RA, sIL13RA2) and three allergy-related transcription factors (FOXP3, STAT3, STAT6) using serum specimens from the Janus Serum Bank Cohort in Oslo, Norway. Blood donors subsequently diagnosed with glioma (n = 487) were matched to controls (n = 487) on age and date of blood draw and sex. We first estimated individual effects of the 12 serum proteins and then interactions between IL4 and IL13 and their receptors using conditional logistic regression. We next tested equality of case-control inter-correlations among the 12 serum proteins. We found that TGFB2 is inversely related to glioblastoma (Odds Ratio (OR) = 0.87, 95% Confidence Interval (CI)) = 0.76, 0.98). In addition, ≤ 5 years before diagnosis, we observed associations between IL4 (OR = 0.82, 95% CI = 0.66, 1.01), sIL4RA (OR = 0.80, 95% CI = 0.65, 1.00), their interaction (OR = 1.06, 95% CI = 1.01, 1.12) and glioblastoma. This interaction was apparent > 20 years before diagnosis (IL4-sIL4RA OR = 1.20, 95% CI = 1.05, 1.37). Findings for glioma were similar. Case correlations were different from control correlations stratified on time before diagnosis. Five years or less before diagnosis, correlations among case serum proteins were weaker than were those among controls. Our findings suggest that IL4 and sIL4RA reduce glioma risk long before diagnosis and early gliomagenesis affects circulating immune function proteins.

## Introduction

Glioma is a heterogeneous primary malignant brain tumor with a median survival time, for the most common adult subtype, glioblastoma, of only 14 months [1]. The absence of treatment that insures long term survival and the brief duration of preclinical symptoms, make it essential that both risk factors for and preclinical evidence of this tumor be identified. Given first, the inverse association between self-reported allergy, asthma [2], prediagnostic serum IgE [3] and glioma and, second, the fact that glioblastoma-initiating cells inhibit T-cell growth and increase proliferation of immune suppressive regulatory T cells [4], we undertook a study of prediagnostic serum immune function proteins to determine whether they affect glioma risk or would indicate early gliomagenesis. Such information may eventually allow prevention, earlier diagnosis or a better understanding of gliomagenesis.

Cytokines control immune reactions related to glioma and its microenvironment. Although there are no known empirical studies of associations between prediagnostic serum cytokines and glioma, there are numerous experimental studies of cytokine expression in glioma tissue and the tumor microenvironment [5]. In addition, there are observational studies of cytokine levels in the peripheral circulation of glioma patients [6, 7]. We identified seven cytokines from previous glioma [5–7] or allergy literature [8, 9] (IL4, IL13, IL5, IL6, IL10, IFNG and TGFB2) to determine whether, they were associated with glioma before diagnosis. We also included two soluble cytokine receptors (sIL4RA [10] and sIL13RA2 [11]) and an exploratory component consisting of three allergy-related transcription factors (FOXP3 [12], STAT3 [13] and STAT6 [14]). Although transcription factors are intracellular proteins and therefore would not normally be found in serum, they may be released into the serum in response to pathological conditions resulting in cell death [15, 16]. For example, Chaung et al. [17] report that, in response to hemorrhagic shock, mitochondrial transcription factor A can be measured in the serum of rats.

Cytokines work in concert [18, 19] therefore their analysis should allow for this synergy [20, 21]. Wu et al. [21] constructed a complex mathematical model of intercellular signaling networks in early stage glioma development. Their model predicts initially strong correlations among cytokines and growth factors in the tumor microenvironment. However, with the onset of rapid tumor growth, most of these correlations are eliminated. Thus, in addition to glioma-control differences in individual circulating serum protein concentration, we evaluated correlations among serum proteins. Our aim was to understand whether there is an association between 12 serum proteins measured before diagnosis and glioma. To achieve this goal, we conducted a nested case–control study using prospectively collected serum samples from the Janus Serum Bank in Oslo, Norway.

## Materials and Methods

### Study Population

The Janus Serum Bank was established in 1972 to conduct epidemiological studies of cancer [22–24]. This biobank is now owned by the Cancer Registry of Norway and contains serum samples from approximately 167,000 men and 158,000 women. Approximately 90% of the serum donors were participants in routine cardiovascular health examinations conducted by the National Health Screening Services. In addition to physical examinations, blood was drawn to evaluate cholesterol and lipid levels. Residual volumes of these samples were stored in the Janus Serum Bank. The majority of these donors were between ages 35 and 49 years old at the time of blood donation. In addition, approximately 10% of the serum samples came from male and female Red Cross Blood Bank donors. Most of these donors were between ages 20 and 65 years old at the time of their blood donation. Samples were stored at −25°C and underwent one thaw–freeze cycle in preparation for the present study.

The final data set contained no personal identifiers. However, initially, personal identification numbers were used to link Janus Serum Bank project blood donors to the Cancer Registry of Norway. We analyzed serum samples from 512 blood donors who were subsequently diagnosed with glioma (International Classification of Disease, Oncology, Third Edition [ICD-O-3] morphology codes 9380–9411, 9420–9480, and 9505) between January 1, 1974 and December 31, 2007. However, we subsequently excluded 13 case participants diagnosed with medulloblastoma or primitive neuroectodermal tumor (ICD-O-3 codes 9470–9474) or pilocytic astrocytoma (ICD-O-3 code 9421) because of their small number together with differences in age distributions of these tumors [25] compared with those of the other glioma participants. Of the remaining 499 case participants, 12 were excluded because they did not have a matching control leaving 487 cases with glioma, 315 of them with glioblastoma (ICD-O-3 morphology code 9440).

A control participant for each glioma case was randomly selected, according to an incidence density sampling scheme, from among blood donors. Controls were individually matched to cases on date of blood collection (±3 months), date of birth (±1 year), county of residence at blood collection and gender. Matched control participants were required to be alive at the date of diagnosis of the case to which they were matched and free from any cancer except non-melanoma skin cancer. In addition, to save valuable serum for use in subsequent biobank studies, potential controls diagnosed with rare tumors (i.e., all tumors other than breast, prostate, and colorectal) after the corresponding case’s date of glioma diagnosis were rejected from the study. Of the 506 control subjects whose serum samples were analyzed 19 were not included in the study because there was no serum from the corresponding glioma case to which they were matched, leaving a total of 487 controls.

### Ethics Statement

The research plan on which the present study is based was approved by the Regional Ethics Committee of Southern Norway and the Norwegian Data Protection Authority. During the Janus Serum Bank’s first years, 1973–1992, donors gave broad verbal consent for use of samples in “cancer research” [26]. No samples were collected from 1993 to 1996. Samples from 1997 and later were collected in conjunction with an explicit informed consent document (Act Relating to Biobanks, § 12, http://ec.europa.eu/research/biosociety/pdf/norwegian_act_biobanks.pdf). These signed forms are stored either at the Cancer Registry of Norway or the Norwegian Institute of Public Health. The Norwegian Data Protection Authority (https://www.datatilsynet.no/English/) has approved of the use of the Janus data and biological samples collected during the period 1972–2004, while requiring that participants that blood donors are free to unconditionally withdraw their consent at any time. Upon withdrawal, their serum samples will be destroyed and associated data deleted (Act Relating to Biobanks, § 14, http://ec.europa.eu/research/biosociety/pdf/norwegian_act_biobanks.pdf). As additional participant protection, all research projects using specimens from the Janus repository and data from the Cancer Registry of Norway need approval from a Regional Committee for Medical and Health Research Ethics. Donors are informed about ongoing research projects through the Cancer Registry web pages (http://www.kreftregisteret.no/en/Research/About-our-Research/).

### Cytokine Microarray Analysis: RayBio Human Cytokine Antibody Array Kits

Cytokine array kits, consisting of a combination of two Human Cytokine Antibody Arrays (G2000, n = 174 and G4000, n = 274) from RayBiotech, Inc. (Norcross, Georgia) were used to measure 278 serum cytokines, soluble cytokine receptors and transcription factors. These array kits were mailed to Professor Eivind Hovig's Laboratory at Oslo University, Norway where serum samples were randomly assigned to print batches. The antibody- based microarray assay is analogous to a sandwich ELISA assay using two sets of anti-cytokine or transcription factor antibodies. The hybridized arrays were scanned for fluorescence using the Agilent scanner G2505C. The scans were obtained with photomultiplier tube settings first at 100% of maximal intensity. If spots were saturated (meaning reaching the maximum 16 bit gray scale level), this would lead to loss of linearity of saturated spots. Several spots were saturated and were rescanned at 30 pmt to prevent spot saturation. Some of these rescans at 30 pmt failed, due to an attempt to remove high background signals, through a washing procedure. This washing procedure generated more background. For these, the 100 pmt scan was used. However, this procedure was applied to few slides, and resulted in a very low level of saturated spots. Tiff images were made from these scans. The Tiff images were segmented using GenePix 6.0, *i*.*e*. converted from image spots to numerical values of grey scale levels per spot. The GenePix result files were read into the statistical programming language R. The "F532 Median" column was chosen as signal without background subtraction, i.e. using median grey level values per spot. A clear batch effect was observed, probably due to different print batches. Replicate spots were subsequently averaged. Ninety-five samples were analyzed twice in different batches (one was analyzed in three different batches) and these values were also averaged.

### Statistical Methods

This article is the first of two analyzing associations among prediagnostic serum protein levels and glioma. In the present paper we were especially interested in the period near the time of diagnosis because we wished to determine whether the early tumor affects immune function serum proteins. However, small samples (*e*.*g*., 55 glioma cases, 55 controls) typically increase the risk of false positive findings [27]. That is, they are more likely to yield statistically significant results when the null hypothesis actually holds than are large samples. However, Wacholder et al. show that when associations for which there is prior evidence are tested in small samples, the probability of false positive findings is reduced. We therefore restricted this initial analysis to a group of 12 allergy and glioma-related serum proteins which, based on previous literature [5, 8, 9], have the highest *a-priori* probability of being associated with glioma.

We first compared the case and control distributions of matching variables (i.e., sex, age and date of blood collection) by inspection in the total data set and among participants whose blood was drawn ≤ 5 years before diagnosis. In subsequent analyses, controls were assigned the date of diagnosis of the case to which they were matched.

To evaluate quality control we estimated the median coefficient of variation and the interquartile range (IQR) of each serum protein that was measured in more than one batch (n = 95) by case status. Samples were randomly assigned to batches independently of their case status (which was not known by the laboratory personnel). We used the Chi-Square and Fisher’s Exact Test to compare the batch distribution by cases and controls.

We next minimized the potential influence of outliers by transforming serum protein values to a natural log scale and then standardizing them to a mean of zero and standard deviation of one. If outliers still affected the results, we replaced serum protein values with their ranks.

To determine whether each prediagnostic serum protein was independently associated with glioblastoma or glioma, we used conditional logistic regression models, conditioned on matched set or batch and stratified on time before diagnosis (All times, ≤ 5, >15 years). In addition, based on prior knowledge [8, 9], we used separate regression models to evaluate interactions between IL4 and IL13, the central allergy cytokines and their receptors.

In the remaining analyses we regard the 12 serum proteins as components of a biological system 21]. We therefore evaluated correlations among them in case-control groups or matrices stratified on time before diagnosis. To visualize associations among these proteins, we first graphed separate glioma and glioblastoma case and control Pearson correlation matrices by time before diagnosis (All times, ≤ 5, > 15). (Results using Spearman rank correlations were similar but are not shown.) We next tested the equality of case and control correlation matrices by time before diagnosis (All times, ≤ 5, > 10, > 15, > 20 years) using the Jennrich test (16).

To find the relative magnitude of case and control correlation coefficients, we added all the absolute values of case correlation coefficients and did the same for the absolute values of control correlations. We then subtracted the case sum from the control sum. Next, we created 1000 bootstrap samples for each of the five time categories and averaged their case-control absolute correlation sum differences. If the sum of absolute values of control correlations was larger than the sum of case correlations, then the difference of the sums would be positive. If case correlation coefficients were larger, then the sum difference would be negative.

To identify individual serum proteins that were driving case-control differences, we calculated absolute differences between case and control correlation coefficients for each serum protein by time before diagnosis. All analyses were conducted using SAS statistical software, version 9.3 (SAS Institute Inc, Cary, NC) or the R language and environment (R Core Team (2013). R: A language and environment for statistical computing. R Foundation for Statistical Computing, Vienna, Austria. (URL http://www.R-project.org/)).

## Results and Discussion

### Characteristics of Study Participants

Table 1 shows the success of the matching scheme. Both glioblastoma and glioma cases and controls are virtually identical with respect to the matching variables (age of and date at blood draw and sex). Participants were relatively young when their blood was collected (*e*.*g*., for total glioma the median age = 42 years, (IQR = 40, 43 years). Therefore, participants diagnosed with glioma whose blood was drawn ≤ 5 years before diagnosis were predictably younger at diagnosis (median age at diagnosis = 45 years, IQR = 43, 48 years) than were those in the total sample (median age at diagnosis = 57 years, IQR = 51, 63 years). The prediagnostic period under investigation is relatively long with a median time between blood collection and date of diagnosis of 15 years (IQR = 9, 21 years).

**Table 1:** Demographic and temporal variables that characterize glioblastoma and glioma case and control study participants^a^.

Variable	Cases	Controls
**Glioblastoma**		
Number	315	315
Percent men	72 (67, 77)^b^	72 (67, 77)
Median age at blood collection	42 (40, 44)^c^	42 (40, 44)
Median age at glioma diagnosis	58 (53, 66)	—-^d^
Median time in years from blood collection to diagnosis	16 (10, 22)	—-
Median date of blood collection	1985 (1975, 1989)	1985 (1975, 1989)
Median date of birth	1943 (1931, 1947)	1943 (1931, 1947)
**Glioblastoma ≤ 5 Years before Diagnosis**		
Number	22	22
Percent men	73 (53, 93)	73 (53, 93)
Median age at blood collection	42 (41, 46)	42 (40, 47)
Median age at glioma diagnosis	45 (44, 50)	—-
Median time in years from blood collection to diagnosis	3 (2, 4)	—-
Median date of blood collection	1986 (1973, 1989)	1986 (1974, 1989)
Median date of birth	1942 (1929, 1947)	1941 (1928, 1947)
**Total Glioma**		
Number	487	487
Percent men	67 (63, 71)	67 (63, 71)
Median age at blood collection	42 (40, 43)	42 (40,43)
Median age at glioma diagnosis	57 (51, 63)	—-
Median time in years from blood collection to diagnosis	15 (9, 21)	—-
Median date of blood collection	1986 (1976, 1989)	1986 (1976, 1989)
Median date of birth	1945 (1932, 1947)	1945 (1933, 1948)
**Total Glioma ≤ 5 Years before Diagnosis**		
Number	55	55
Percent men	58 (45, 72)	58 (45, 72)
Median age at blood collection	42 (41, 46)	42(41,47)
Median age at glioma diagnosis	45 (43, 48)	—-
Median time in years from blood collection to diagnosis	3 (1,4)	—-
Median date of blood collection	1988 (1983, 1990)	1987 (1984, 1990)
Median date of birth	1946 (1933, 1948)	1946 (1932, 1948)

a. Case study participants were blood donors (1974–2007) to the Janus Serum Bank, Oslo, Norway, who were subsequently diagnosed with glioblastoma or other types of glioma. Control participants were individually matched to case participants on 2-year age interval, date of blood collection and sex.

b. 95% confidence interval

c. Interquartile range

d. Not applicable

### Coefficients of Variation in Duplicate Samples

Duplicate samples (47 glioma cases, 48 controls) were measured in different batches and the median coefficients of variation were calculated for each serum protein. S1 Table shows that all coefficients of variation were .11 or less except for those for IL5 and sIL13RA. The average of the coefficient of variation medians over all 12 serum proteins is .09 for cases and the same value for controls.

### Case-Control Distributions by Batch

As a result of random assignment of serum samples to batch, batches were evenly distributed between all cases and controls S2 Table (Chi-Square (12*df*) = 1.95; *P* = 1.00). In addition, batches were evenly distributed among cases and controls in the subset whose blood samples were collected ≤ 5 years before glioma diagnosis (Fisher’s Exact Test; *P* = .99). Furthermore, conditioning the logistic regression models, discussed below, on batch did not affect our findings.

### Rescanned Samples

Samples were not assigned to rescanning at random (See Cytokine Microarray Analysis above), however, the random assignment of samples to batch resulted in the equal distribution of rescanned samples among cases and controls (30 glioma cases, 29 controls; Chi-Square (1*df*) = 0.02, *P* = .89; 21 glioblastoma cases, 21 controls; Chi-Square = 0.00, *P* = 1.00). The hypothesis of equal distribution of rescanned samples among cases and controls was not rejected.

### Associations between Individual Cytokine Concentration and Glioblastoma and Glioma

When all glioblastoma cases (n = 315) and their controls (n = 315) were included in the analysis, only TGFB2 was statistically significantly associated with glioblastoma (OR = 0.87, 95% CI = 0.76, 0.98). S1 Fig shows a graph of TGFB2 against time before diagnosis during the ten years prior to diagnosis. This graph suggests a diminution of TGFB2 concentration among glioblastoma cases, but not among their matched controls, as the time of diagnosis approaches.

Other than TGFB2, none of the 11 serum proteins was independently related to total glioma or glioblastoma or either tumor within the strata of time before diagnosis. In the context of these negative results, we must consider the TGFB2 finding with skepticism because it was the only statistically significant test (*P* < .05*)* among the 72 that we conducted (12 serum proteins tested separately for their association with glioma and glioblastoma for three time periods). We would expect approximately four of these tests to be false positives; however, we only found one significant result. In addition, Wu et al. [21], whose glioma model evaluates prediagnostic correlations among cytokines, suggest that individual cytokine concentration at one point in time may not be informative in a dynamic system and may even be misleading. They argue that in a signaling system it is the interaction among the signaling cytokines that is of central importance which may not be related to their concentration.

### TGFB2 Literature

In spite of our doubts of the validity of the TGFB2 finding, it is worthwhile to examine previous literature on its association with glioma because, as previously noted in the Statistical Methods Section above, the higher the prior probability of a finding, the less likely it is to be a false positive. TGFB2 was initially labeled “glioblastoma-T-cell suppressor factor” due to its apparent role in glioma progression. In fact, modulation of this cytokine has been proposed as a goal of glioblastoma treatment [28]. However, in an article entitled, “TGF-B: Duality of Function between Tumor Prevention and Carcinogenesis”, Principe et al. [29] present evidence of tumor suppressor activities of TGFB in early stage carcinogenesis.

### Interaction between IL4 and sIL4RA

**Fig 1:**
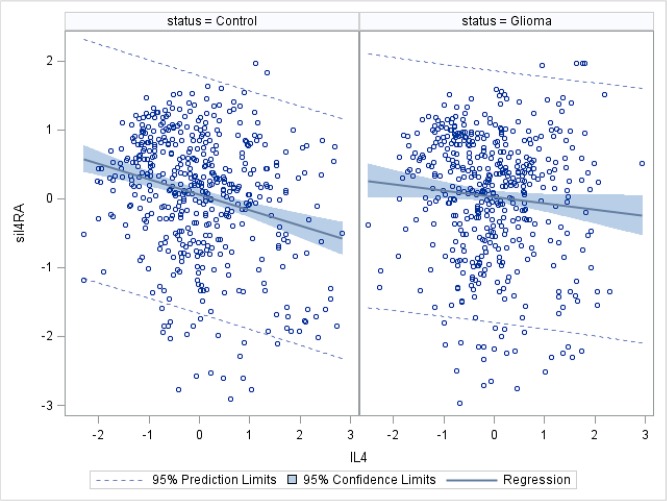
Association between IL4 and sIL4RA among glioma cases and controls (Observations more than three standard deviations from the mean are excluded). Graph on left shows the association among glioma controls (n = 482); graph on right shows the association among glioma cases (n = 474).

Fig 1 shows a stronger inverse association between the standardized logs of IL4 and sIL4RA among controls than among cases indicating an interaction by case status. Outliers more than three standard deviations from the mean were excluded from the graph to avoid their undue their influence. The case-control interaction is stronger when all values are included (S2 Fig) however it is not possible to determine which figure (Fig 1 or S2 Fig) accurately represents the true IL4 sIL4RA association. Therefore, to be conservative, we included all values, as in S2 Fig, but transformed them to ranks. The data were then stratified on time before diagnosis. In Table 2, the positive interaction term indicates that IL4’s inverse association with glioblastoma and glioma is reduced over levels of sIL4RA (and *vice versa*). That is, the observed negative effects of IL4 and sIL4RA on these tumors are not as negative as their main effects alone would suggest. Using the same statistical models as those in Table 2, we found no evidence of interactions among IL13 and the sIL4RA or sIL13RA2 receptors.

**Table 2:** Association between ranks^a^ of IL4, sIL4RA, their interaction and glioblastoma and glioma.

	Odds Ratios (95% Confidence Intervals	
Time from blood collection to tumor diagnosis^b^	Glioblastoma	Total Glioma
*All study participants*, *n (cases/controls)*	315/315	487/487
IL4	0.82 (0.66, 1.01)	0.90 (0.80, 1.00)
sIL4RA	0.80 (0.65, 1.00)	0.86 (0.76, 0.97)
IL4-sIL4RA	1.06 (1.01, 1.12)	1.02 (1.01, 1.04)
*≤ 5 years before diagnosis*, *n*	22/22	55/55
IL4	0.73 (0.35, 1.50)	0.77 (0.54, 1.10)
sIL4RA	0.71 (0.30, 1.67)	0.77 (0.51, 1.17)
IL4-sIL4RA	1.07 (0.89, 1.29)	1.04 (0.98, 1.11)
*>10 years before diagnosis*, *n*	242/243^b^	347/347
IL4	0.81 (0.63, 1.04)	0.91 (0.80, 1.04)
sIL4RA	0.75 (0.58, 0.97)	0.84 (0.72, 0.97)
IL4-sIL4RA	1.08 (1.01, 1.15)	1.02 (1.00, 1.05)
*>15 years before diagnosis*, *n*	167/169	228/230
IL4	0.73 (0.55, 0.98)	0.89 (0.77, 1.03)
sIL4RA	0.66 (0.48, 0.92)	0.84 (0.71, 1.00)
IL4-sIL4RA	1.19 (1.03, 1.21)	1.03 (1.00, 1.06)
*>20 years before diagnosis*, *n*	95/96	126/126
IL4	0.65 (0.44, 0.96)	0.81 (0.66, 0.99)
sIL4RA	0.54 (0.31, 0.93)	0.72 (0.52, 0.99)
IL4-sIL4RA	1.02 (1.05, 1.37)	1.06 (1.01, 1.12)

a. Single rank changes were too small to interpret therefore one unit of IL4 andsIL4RA equals 100 ranks.

b. Controls were assigned the date of diagnosis of the case to which they were matched.

### IL4 and sIL4RA Literature

To the extent that IL4 and sIL4RA are components of a complex biological system, the models in Table 2 are overly simple. Nonetheless, the previous literature may assist in understanding the implications of the models Table 2. It has been established that sIL4RA inhibits IL4 [30]. For this reason, it has been proposed that this soluble receptor be used to treat allergic conditions [31]. Therefore, the positive value of the IL4-sIL4RA interaction term is consistent with the allergy-glioma hypothesis in that blocking IL4, an important allergy cytokine, increases the risk of glioma. Unfortunately, the association between this interaction and glioma is more complex in that sIL4RA activates IL13 [30], another cytokine central to allergy. Also a problem in interpreting our models in the context of allergy is that we did not measure expression of membrane-bound (memIL4RA), a mediator of IL4, which could confound the IL4-sIL4RA association. That memIL4RA receptor may participate in glioma progression is suggested by Schwartzbaum et al.’s [32] finding that expression of the memIL4RA receptor in glioblastoma tissue is inversely related to a measure of tumor aggression (CD133). Nestor et al [33] report that sIL4RA concentration is inversely related to expression of memIL4RA, however, the interpretation of this result in the context of allergy and glioma would depend on whether IL4 was bound to sIL4RA. This literature confirms the complex processes underlying a potential association between IL4, its receptors and glioma risk.

### Graphs of Case and Control Correlation Matrices by Time before Diagnosis

**Fig 2:**
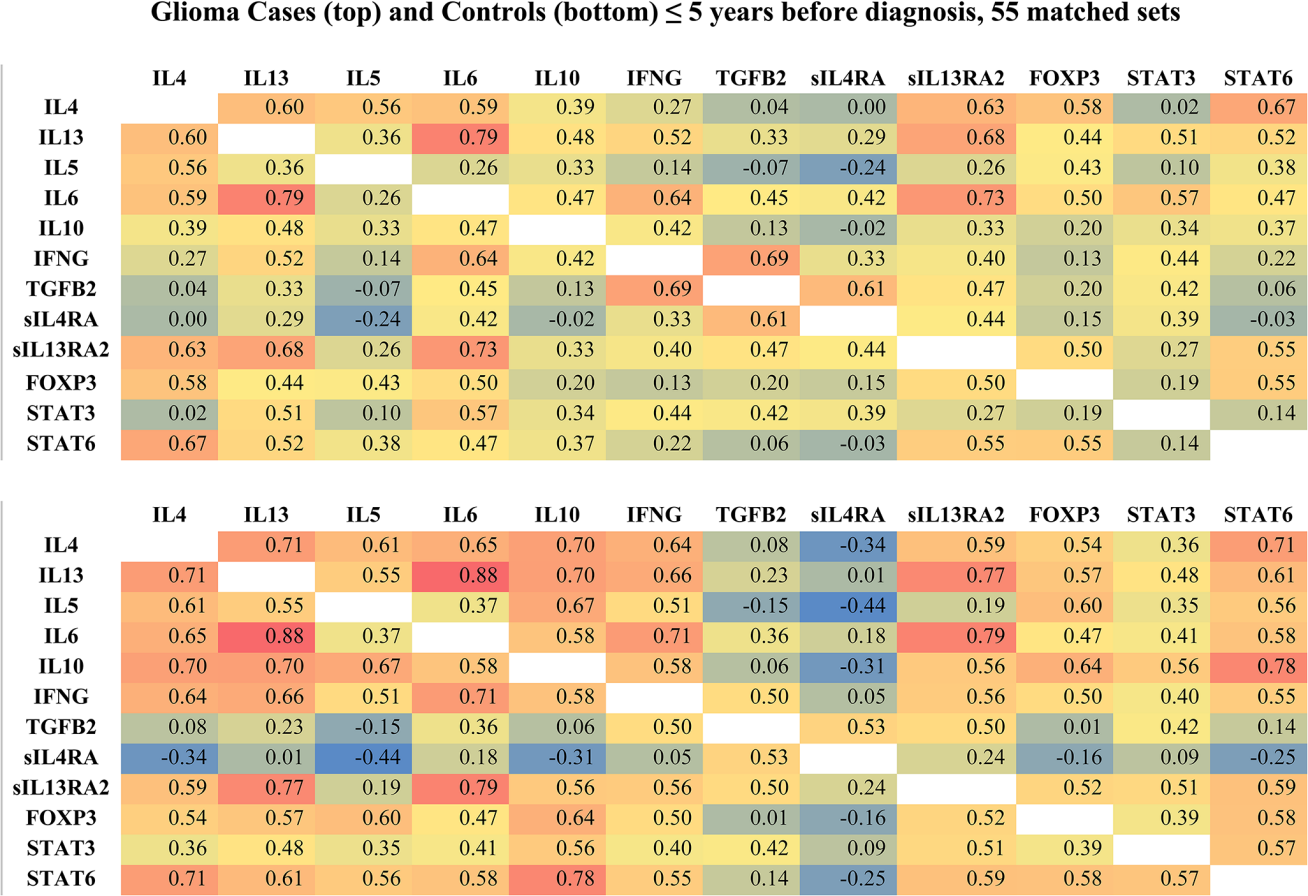
Glioma Case and Control Serum Protein Pearson Correlation Matrices ≤ 5 Years before Diagnosis. The top graph shows case correlations, the bottom graph shows control correlations. Color scale: blue = negative correlations, green, yellow = moderate correlations, red = positive correlations.

**Fig 3:**
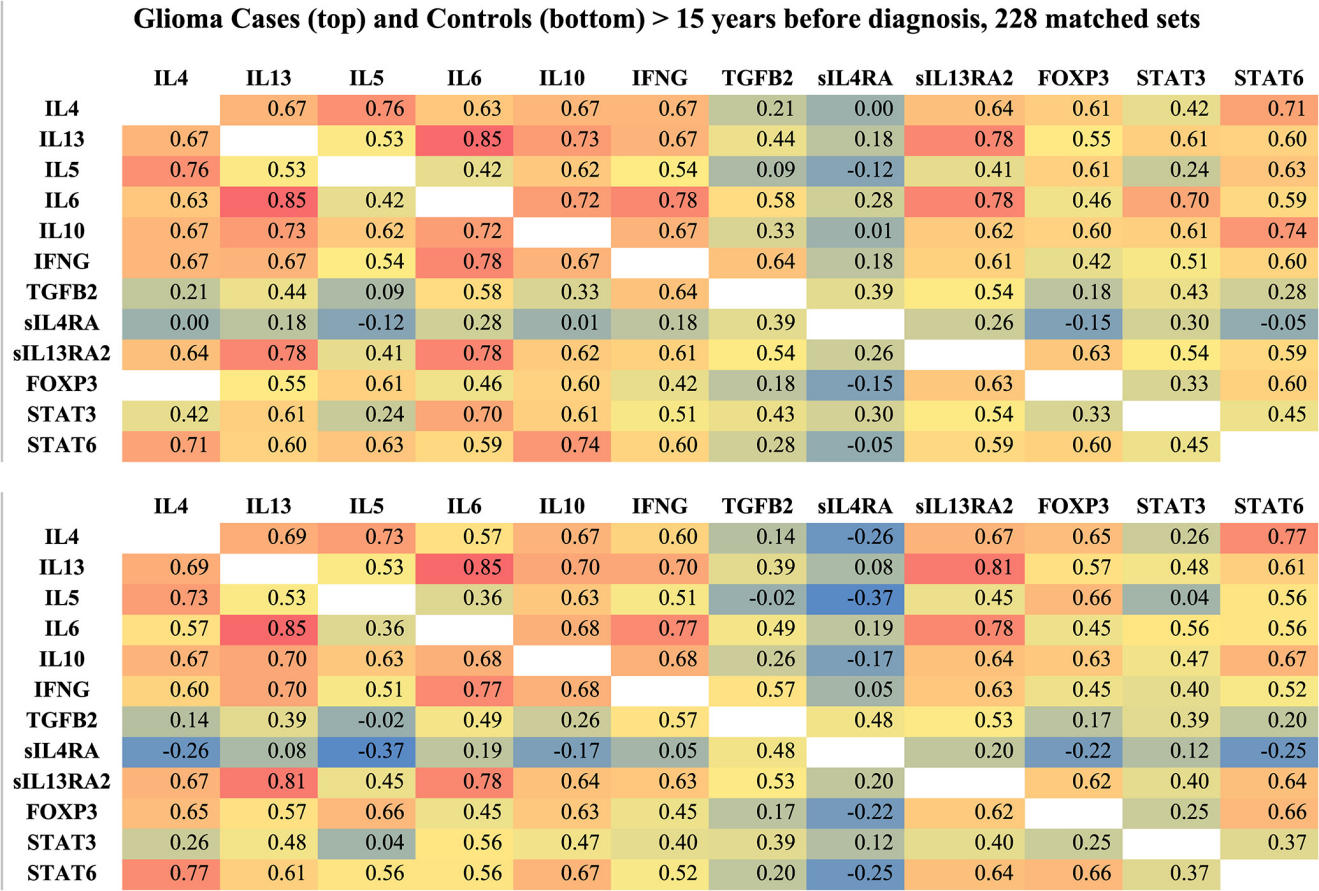
Glioma Case and Control Serum Protein Pearson Correlation Matrices > 15 Years before Diagnosis. The top graph shows case correlations, the bottom graph shows control correlations. Color scale: blue = negative correlations, green, yellow = moderate correlations, red = positive correlations.

In Fig 2 the intensity of color indicates the strength of the correlations, with red showing strong positive and blue strong negative correlations. In this figure, ≤ 5 years before diagnosis, control correlation coefficients (bottom graph) are, in general, further from the null than are case correlation coefficients (top graph). In particular, negative correlations of serum proteins with sIL4RA are stronger among controls than among cases. In Fig 3, >15 years before diagnosis, while case-control correlation coefficient differences persist for sIL4RA, overall case and control correlation differences are smaller than in Fig 2 (≤ 5 years before diagnosis).

### Statistical Tests of Equality of Case and Control Correlation Matrices

All tests of equality of case-control correlation matrices, stratified on five levels of time before diagnosis, were highly statistically significant (*P* < .0001) for both glioma and glioblastoma. However, this statistic was designed to test the equality of correlation matrices and not the direction of their differences. Furthermore, this result does not provide information as to whether differences between case-control correlation matrices are primarily attributable to observed correlations with sIL4RA or a more general inequality.

### Differences between Case and Control Sums of Absolute Values of Correlation Coefficients

To quantify the apparent visual difference between case and control correlation coefficients that we observed in Fig 2, we added all case correlations and all control correlations and compared their sums. We used absolute rather than actual values because we were interested in the distance of correlations from zero and a correlation of -0.70 is the same distance from zero as a correlation of 0.70. A positive value of the difference of case from control sums in Table 3 indicates that control correlation coefficients are further from the null and a negative value suggests that case correlations are further from the null.

**Table 3:** Differences between case and control sums of absolute correlations by time before diagnosis.

	Difference between Sums of Absolute Values of Correlation Coefficients (95% Confidence Intervals)	
Time from blood collection to tumor diagnosis^a^	Glioblastoma	Total Glioma
*All study participants*, *n (cases/controls)*	315/315	487/487
Difference between sums (95% CI)	0.46^b^ (-3.78, 4.77)^c^	-1.49^d^ (-4.69, 1.75)
*≤ 5 years before diagnosis*, *n*	22/22	55/55
Difference between sums (95% CI)	3.73 (-9.86, 15.38)	5.50 (-3.36, 14.11)
*>10 years before diagnosis*, *n*	242/243^e^	347/347
Difference between sums (95% CI)	-1.20 (-5.86, 3.51)	-2.28 (-6.17, 1.28)
*>15 years before diagnosis*, *n*	167/169	228/230
Difference between sums (95% CI)	-1.02 (-6.55, 4.46)	-1.36 (-5.84, 3.13)
*>20 years before diagnosis*, *n*	95/96	126/126
Difference between sums (95% CI)	-1.61 (-9.10, 5.51)	-0.56 (-6.43, 5.04)

a. Controls were assigned the date of diagnosis of the case to which they were matched.

b. Difference greater than 0.00 indicates absolute values of control correlations are larger than those of cases.

c. If the 95% confidence interval includes zero then its corresponding p-value is not statistically significant.

d. Difference less than 0.00 indicates absolute values of case correlations are larger than those among controls.

e. Controls are matched to cases within three months of the time of blood draw. Therefore a matched pair may fall into separate time categories thus accounting for unequal numbers in time category.

Table 3 shows that ≤ 5 years before diagnosis, consistent with our visual impression of Fig 2, control coefficients are further from the null than are case coefficients. More than ten years before diagnosis, case correlation coefficients appear to be further from the null, however, these negative differences are small, non-significant and may therefore represent sampling variation around approximately equal case and control correlation distances from the null.

Paradoxically, while we have indicated that these serum proteins interact and should therefore be treated as a system, our analysis implicitly assumes independence of individual correlation coefficients (*i*.*e*., the correlations are not confounded by each other nor do they interact). That is, if correlation coefficients do not represent true associations between variables it may not be meaningful to add them. The assumption of validity of the correlation coefficients is a first step in understanding the effects of the preclinical tumor on these serum proteins and is made for purposes of quantifying our visual impressions of Figs 2 and 3.

### Previous Literature on Correlations among Prediagnostic Cytokines and Cancer

Our findings of diminished case correlations ≤ 5 years before diagnosis are consistent with those predicted Wu et al. [21] who constructed an empirically-based mathematical model of intercellular signaling in both the microenvironment and tumor cells from early gliomagenesis to the time of rapid tumor growth. Fifteen cytokines and growth factors were among the signaling constituents in their model (including IL6, IL10 and TGFB which are also analyzed in the present study). Their model predicts that correlations among these 15 cytokines disappear as the tumor initiates rapid growth. This prediction is consistent with our finding of the weakening of the case cytokine correlation structure prior to tumor diagnosis. However, the timing of changes in cytokine inter-correlations predicted by their model differs from that we observed. That is, we find changes in the case cytokine correlation structure ≤ 5 years before diagnosis with the median time being three years before diagnosis (IQR = 1,4 years). It is therefore unlikely that the tumors in our study have entered a stage of rapid development. However, although their model is empirically based, not all the initial values for the model parameters were known. These authors write that these unknown parameters would “only change the quantitative time line” thus possibly accounting for differences between the time of weakening of the correlation structure predicted by their model and our results. A further discrepancy between their model and our study is that they modeled interactions among signaling networks in the tumor microenvironment and the tumor, while we analyzed cytokines in the peripheral circulation. In addition, we find a prediagnostic weakening of the correlation structure; they find that correlations among cytokines disappear. Finally, 12 of the 15 cytokines they included in their model are not included in the present study.

Also consistent with the prediagnostic weakening of case cytokine correlation structure is evidence presented by Bartee and McFadden [34] showing that several types of cancer cells have lost the ability to induce synergy between the antiviral cytokines TNF and IFNB. In a review of cytokine synergy and its role in anti-viral immunity [20] they suggest that escape from the synergistic effects of cytokines may be a step in carcinogenesis.

### Graphs of Absolute Differences between Case and Control Correlations

Next, to facilitate visual comparison of case and control correlation matrices in Figs 2 and 3 and determine whether there are individual serum proteins that may be driving case-control differences, we graphed the absolute difference between case and control correlations for each serum protein. For example, if the case correlation was-.70 and the control correlation was .70 the difference would be -1.4, however to indicate this difference is of the same magnitude as that between case and control correlations of .70 and-.70 we excluded the sign.

**Fig 4:**
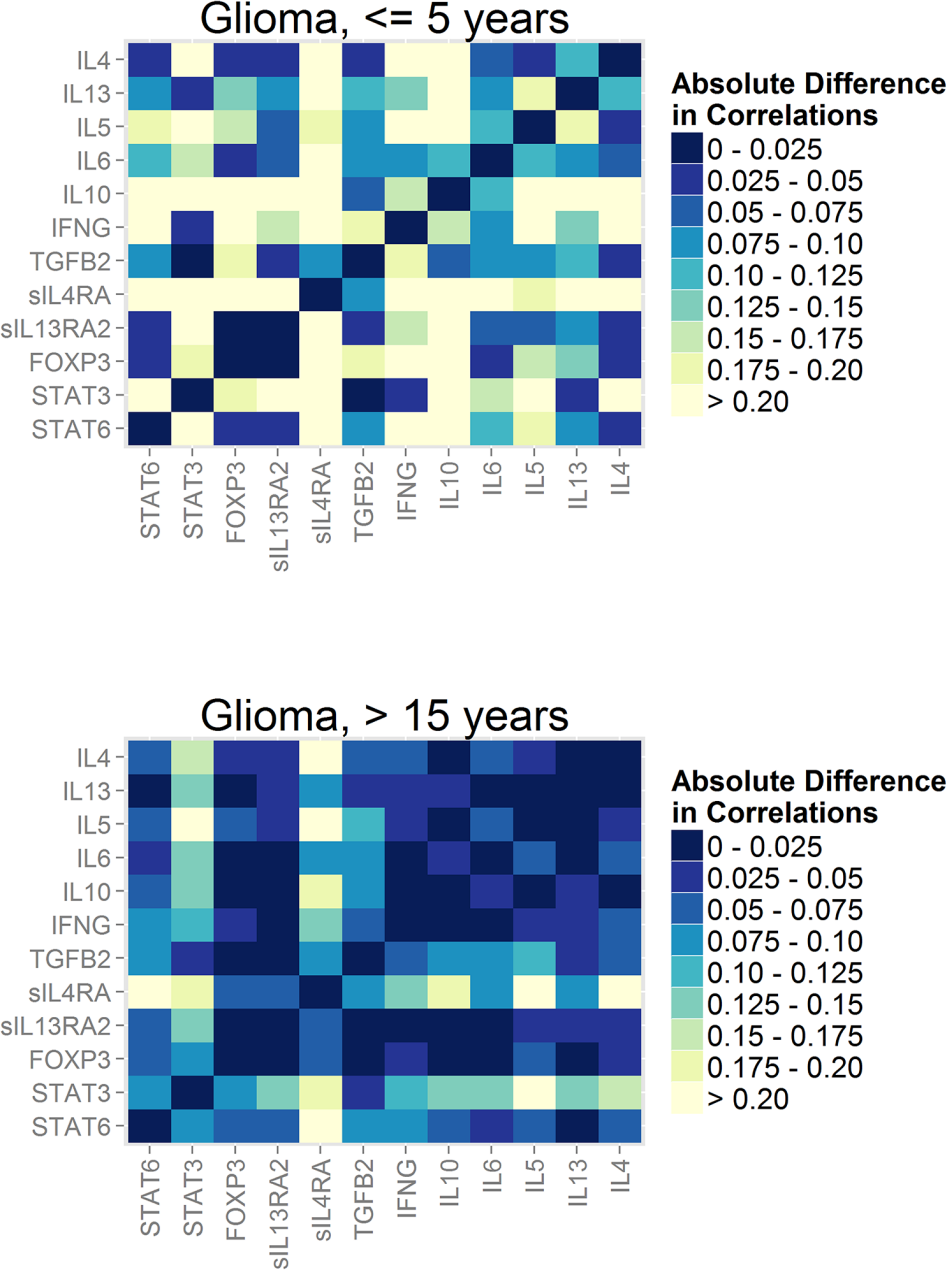
Absolute difference between glioma case and control correlation coefficients. Lighter colors indicate larger absolute differences between case and control correlation coefficients. Top graph represents glioma cases and controls whose blood was drawn ≤ 5 years before diagnosis (n = 55 cases, 55 controls). Bottom graph represents glioma cases and controls whose blood was drawn > 15 years before diagnosis (n = 228 cases and 230 controls).

In Fig 4, lighter colors indicate larger absolute differences. The salient feature of this figure is the number of large case-control differences in the top graph (≤5 years before glioma diagnosis) compared to those in the bottom graph (>15 years before diagnosis). In addition, > 15 years before diagnosis (bottom graph), case-control differences are largest for correlations involving sIL4RA (with IL4, IL5, IL10, STAT3 and STAT6) and STAT3 (with IL4, IL13, IL5, IL6, sIL4RA and sIL13RA2). These patterns are also apparent for glioblastoma (S3 Fig).

### STAT3 Literature

The relationship between STAT3 in serum and its function as a transcription factor is unknown; however intracellular STAT3 has opposing effects on gliomagenesis. While there is extensive evidence of participation of STAT3 in gliomagenesis and progression [35, 36], this cytokine may also suppress glioblastoma depending on the mutation profile of the tumor [37].

### Summary

In the first known study of associations among prediagnostic serum cytokines and glioma, we identified inverse associations between TGFB2 and glioblastoma and between IL4, sIL4RA and both glioma and glioblastoma. Negative associations between IL4 and sIL4RA and these tumors are slightly reduced by their positive interaction. Both the main effects and their interaction are statistically significant > 20 years before diagnosis suggesting that they alter tumor risk. Furthermore, five tests of equality of case and control correlation matrices stratified on time before diagnosis were rejected. In addition, while the correlation structure of cases weakens ≤ 5 years before diagnosis that of controls does not. More than 15 years before diagnosis, absolute differences between glioma and control correlations are largest for correlations with sIL4RA and STAT3. These differences are similar for glioblastoma.

### Limitations

The major limitation of the present study is that the observations closest to the time of diagnosis (≤ 5 years), in which we found the largest case-control correlation differences, are based on a relatively small sample (glioma = 55 matched sets, glioblastoma = 22 matched sets). Therefore it is possible that our statistically significant findings of case-control correlation differences are false positives. However, the prediagnostic weakening of the correlation structure is consistent with predictions of an empirically based mathematical model of gliomagenesis [21]. As this model predicts, we found a pattern of case-control correlation differences, but only one serum protein concentration difference. Furthermore, due to interrelationships among the 12 selected serum proteins and the relatively small sample, we cannot identify causal correlations or networks but rather must consider trends in the correlation matrices as a whole together with correspondence between our findings and those in the previous literature. Finally, it is possible that results for serum transcription factors actually represent non-specific binding by antibodies for FOXP3, STAT3 and STAT6. The validity of associations between these transcription factors, cytokines and glioma can be examined in subsequent studies.

### Blood-brain barrier and serum cytokines

Cytokines regulate local intra- and intercellular immune function and due to their strong affinities with their receptors are produced in small amounts (picograms per milliliter). It may therefore seem unlikely that serum cytokines would reflect those produced in the brain during early stages of gliomagenesis. However, recent research suggests communication between the brain and the peripheral immune system [38–40]. An example of this interaction is the fact that endothelial cells that constitute the blood-brain barrier secrete cytokines that are released into the peripheral circulation [41]. In addition, inflammation in the brain elicits a response from peripheral cytokines [40]. Whether cytokines that we observed in the prediagnostic serum are those directly produced by the tumor or its microenvironment or represent systemic responses to gliomagenesis is not essential for the validity of our study. Rather, one of our goals was to identify cytokines that may be altered by early glioma development whatever their source.

## Conclusions

Using prior knowledge and examining correlations among 12 serum proteins, we have identified an interaction between IL4 and sIL4RA and glioma that is present long before tumor diagnosis and may therefore represent a route by which allergy reduces glioma risk. In addition, we found weakening of serum protein correlations among cases but not among controls ≤ 5 years before diagnosis. Assuming our findings can be replicated, whether this serum protein pattern is unique to prediagnostic glioma or can be found before diagnosis in people subsequently diagnosed with other tumors will be determined by subsequent research.

## Supporting Information

S1 Fig Association between TGFB2 and time before diagnosis among glioblastoma cases and controls. Graph is restricted to ten years before diagnosis. Graph on left shows the association among glioma controls (n = 72); graph on right shows the association among glioma cases (n = 73).
(DOC)

S2 Fig Association between IL4 and sIL4RA among glioma cases and controls (All observations). Graph on left shows the association among glioma controls (n = 487); graph on right shows the association among glioma cases (n = 487).
(DOC)

S3 Fig Absolute difference between glioblastoma case and control correlation coefficients. Lighter colors indicate larger absolute differences between case and control correlation coefficients. Top graph represents glioma cases and controls whose blood was drawn ≤ 5 years before diagnosis (n = 22 cases, 22 controls). Bottom graph represents glioma cases and controls whose blood was drawn > 15 years before diagnosis (n = 167 cases and 169 controls).
(DOC)

S1 Table Median coefficients of variation (CV) by serum protein: Based on duplicate values collected in different batches. (DOC)

S2 Table Glioma case-control status by batch number. (DOC)

## References

[1] Koshy M, Villano JL, Dolecek TA, Villano JL, Howard A, Mahmood U, et al Improved survival time trends for glioblastoma using the SEER 17 population-based registries. J Neurooncol 2012;107:207–12. 10.1007/s11060-011-0738-7 21984115 PMC4077033

[2] Zhao H, Cai W, Su S, Zhi D, Lu J, Liu S. Allergic conditions reduce the risk of glioma: a meta-analysis based on 128,936 subjects. Tumour biology: the journal of the International Society for Oncodevelopmental Biology and Medicine. 2013 Epub 2013/12/19. 10.1007/s13277-013-1514-4 . 24347487

[3] Schwartzbaum J, Ding B, Johannesen TB, Osnes LT, Karavodin L, Ahlbom A, et al Association between prediagnostic IgE levels and risk of glioma. J Natl Cancer Inst. 2012;104(16):1251–9. Epub 2012/08/03. djs315 [pii] 10.1093/jnci/djs315 22855780 PMC3424222

[4] Wei J, Barr J, Kong LY, Wang Y, Wu A, Sharma AK, et al Glioma-associated cancer-initiating cells induce immunosuppression. Clin Cancer Res. 2010;16(2):461–73. Epub 2010/01/14. 10.1158/1078-0432.CCR-09-1983 20068105 PMC2943842

[5] Zhu VF, Yang J, Lebrun DG, Li M. Understanding the role of cytokines in Glioblastoma Multiforme pathogenesis. Cancer Lett. 2012;316(2):139–50. Epub 2011/11/15. 10.1016/j.canlet.2011.11.001 . 22075379

[6] Zisakis A, Piperi C, Themistocleous MS, Korkolopoulou P, Boviatsis EI, Sakas DE, et al Comparative analysis of peripheral and localised cytokine secretion in glioblastoma patients. Cytokine. 2007 . 17697783 10.1016/j.cyto.2007.05.012

[7] Lippitz BE. Cytokine patterns in patients with cancer: a systematic review. Lancet Oncol. 2013;14(6):e218–28. Epub 2013/05/04. 10.1016/S1470-2045(12)70582-X . 23639322

[8] Chatila TA. Interleukin-4 receptor signaling pathways in asthma pathogenesis. Trends in molecular medicine. 2004;10(10):493–9. Epub 2004/10/07. 10.1016/j.molmed.2004.08.004 . 15464449

[9] Williams CM, Rahman S, Hubeau C, Ma HL. Cytokine pathways in allergic disease. Toxicologic pathology. 2012;40(2):205–15. Epub 2012/02/04. 10.1177/0192623311430694 . 22301949

[10] Kruse S, Forster J, Kuehr J, Deichmann KA. Characterization of the membrane-bound and a soluble form of human IL-4 receptor alpha produced by alternative splicing. Int Immunol. 1999;11(12):1965–70. Epub 1999/12/11. . 10590262 10.1093/intimm/11.12.1965

[11] Chen W, Sivaprasad U, Tabata Y, Gibson AM, Stier MT, Finkelman FD, et al IL-13R alpha 2 membrane and soluble isoforms differ in humans and mice. J Immunol. 2009;183(12):7870–6. Epub 2009/12/17. 10.4049/jimmunol.0901028 20007572 PMC2822278

[12] Strombeck A, Rabe H, Lundell AC, Andersson K, Johansen S, Adlerberth I, et al High proportions of FOXP3(+) CD25(high) T cells in neonates are positively associated with allergic sensitization later in childhood. Clin Exp Allergy. 2014;44(7):940–52. Epub 2014/02/18. 10.1111/cea.12290 24528482 PMC4215110

[13] Simeone-Penney MC, Severgnini M, Tu P, Homer RJ, Mariani TJ, Cohn L, et al Airway epithelial STAT3 is required for allergic inflammation in a murine model of asthma. J Immunol. 2007;178(10):6191–9. Epub 2007/05/04. . 17475846 10.4049/jimmunol.178.10.6191

[14] Bellinghausen I, Brand P, Bottcher I, Klostermann B, Knop J, Saloga J. Production of interleukin-13 by human dendritic cells after stimulation with protein allergens is a key factor for induction of T helper 2 cytokines and is associated with activation of signal transducer and activator of transcription-6. Immunology. 2003;108(2):167–76. Epub 2003/02/04. 12562325 10.1046/j.1365-2567.2003.01576.x PMC1782882

[15] Poon IK, Lucas CD, Rossi AG, Ravichandran KS. Apoptotic cell clearance: basic biology and therapeutic potential. Nat Rev Immunol. 2014;14(3):166–80. Epub 2014/02/01. 10.1038/nri3607 24481336 PMC4040260

[16] Sangiuliano B, Perez NM, Moreira DF, Belizario JE. Cell death-associated molecular-pattern molecules: inflammatory signaling and control . Mediators of inflammation. 2014;2014:821043 Epub 2014/08/21. 10.1155/2014/821043 25140116 PMC4130149

[17] Chaung WW, Wu R, Ji Y, Dong W, Wang P. Mitochondrial transcription factor A is a proinflammatory mediator in hemorrhagic shock. Int J Mol Med. 2012;30(1):199–203. Epub 2012/04/04. 10.3892/ijmm.2012.959 22469910 PMC3981640

[18] Turrin NP, Plata-Salaman CR. Cytokine-cytokine interactions and the brain. Brain Res Bull. 2000;51(1):3–9. Epub 2000/02/02. . 10654575 10.1016/s0361-9230(99)00203-8

[19] Ksendzovsky A, Glick RP, Polak P, Simonini MV, Sharp AJ, Newman T, et al Mechanisms of Cytokine-Induced Glioma Immunosuppression. The Open Cancer Immunology Journal. 2010;3:30–5.

[20] Bartee E, McFadden G. Cytokine synergy: an underappreciated contributor to innate anti-viral immunity. Cytokine. 2013;63(3):237–40. Epub 2013/05/23. 10.1016/j.cyto.2013.04.036 23693158 PMC3748162

[21] Wu Y, Garmire LX, Fan R. Inter-cellular signaling network reveals a mechanistic transition in tumor microenvironment. Integrative biology: quantitative biosciences from nano to macro. 2012;4(12):1478–86. Epub 2012/10/20. 10.1039/c2ib20044a 23080410 PMC3502715

[22] Jellum E, Andersen A, Lund-Larsen P, Theodorsen L, Orjasaeter H. The JANUS serum bank. Sci Total Environ. 1993;139–140:527–35. Epub 1993/11/01. . 8272856 10.1016/0048-9697(93)90049-c

[23] Jellum E, Andersen A, Lund-Larsen P, Theodorsen L, Orjasaeter H. Experiences of the Janus Serum Bank in Norway. Environ Health Perspect. 1995;103 Suppl 3:85–8. . 7635118 10.1289/ehp.95103s385 PMC1519017

[24] Langseth H, Gislefoss R, Martinsen JI, Stornes A, Lauritzen M, Andersen A, et al The Janus Serum Bank-From sample collection to cancer research Oslo: Cancer Registry of Norway; 2009.

[25] CBTRUS. CBTRUS Statistical Report: Primary Brain and Central Nervous System Tumors Diagnosed in the United States in 2004–2007. Central Brain Tumor Registry of the United States, 2011.

[26] Helgesson G, Dillner J, Carlson J, Bartram CR, Hansson MG. Ethical framework for previously collected biobank samples. Nat Biotechnol. 2007;25(9):973–6. Epub 2007/09/12. nbt0907-973b [pii] 10.1038/nbt0907-973b . 17846619

[27] Wacholder S, Chanock S, Garcia-Closas M, El Ghormli L, Rothman N. Assessing the probability that a positive report is false: an approach for molecular epidemiology studies. J Natl Cancer Inst. 2004;96(6):434–42. . 15026468 10.1093/jnci/djh075 PMC7713993

[28] Hau P, Jachimczak P, Schlaier J, Bogdahn U. TGF-beta2 signaling in high-grade gliomas. Curr Pharm Biotechnol. 2011;12(12):2150–7. Epub 2011/05/31. . 21619538 10.2174/138920111798808347

[29] Principe DR, Doll JA, Bauer J, Jung B, Munshi HG, Bartholin L, et al TGF-beta: duality of function between tumor prevention and carcinogenesis. J Natl Cancer Inst. 2014;106(2):djt369 Epub 2014/02/11. 10.1093/jnci/djt369 24511106 PMC3952197

[30] Andrews AL, Holloway JW, Holgate ST, Davies DE. IL-4 receptor alpha is an important modulator of IL-4 and IL-13 receptor binding: implications for the development of therapeutic targets. J Immunol. 2006;176(12):7456–61. Epub 2006/06/06. . 16751391 10.4049/jimmunol.176.12.7456

[31] Holtzman MJ. Drug development for asthma. American journal of respiratory cell and molecular biology. 2003;29(2):163–71. Epub 2003/07/25. 10.1165/rcmb.F276 . 12878583

[32] Schwartzbaum JA, Huang K, Lawler S, Ding B, Yu J, Chiocca EA. Allergy and inflammatory transcriptome is predominantly negatively correlated with CD133 expression in glioblastoma. Neuro Oncol. 2010;12(4):320–7. Epub 2010/03/24. nop035 [pii] 10.1093/neuonc/nop035 20308310 PMC2940608

[33] Nestor CE, Dadfar E, Ernerudh J, Gustafsson M, Bjorkander J, Benson M, et al Sublingual immunotherapy alters expression of IL-4 and its soluble and membrane-bound receptors. Allergy. 2014;69(11):1564–6. Epub 2014/08/19. 10.1111/all.12505 . 25130266

[34] Bartee E, McFadden G. Human cancer cells have specifically lost the ability to induce the synergistic state caused by tumor necrosis factor plus interferon-beta. Cytokine. 2009;47(3):199–205. Epub 2009/07/31. 10.1016/j.cyto.2009.06.006 . 19640730 PMC4376283

[35] McFarland BC, Hong SW, Rajbhandari R, Twitty GB Jr., Gray GK, Yu H, et al NF-kappaB-induced IL-6 ensures STAT3 activation and tumor aggressiveness in glioblastoma. PLoS ONE. 2013;8(11):e78728 Epub 2013/11/19. 10.1371/journal.pone.0078728 24244348 PMC3823708

[36] Wei J, Barr J, Kong LY, Wang Y, Wu A, Sharma AK, et al Glioblastoma cancer-initiating cells inhibit T-cell proliferation and effector responses by the signal transducers and activators of transcription 3 pathway. Mol Cancer Ther. 2010;9(1):67–78. Epub 2010/01/08. 1535-7163.MCT-09-0734 [pii] 10.1158/1535-7163.MCT-09-0734 20053772 PMC2939737

[37] de la Iglesia N, Puram SV, Bonni A. STAT3 regulation of glioblastoma pathogenesis. Curr Mol Med. 2009;9(5):580–90. Epub 2009/07/16. . 19601808 10.2174/156652409788488739 PMC2712135

[38] Quan N, Banks WA. Brain-immune communication pathways. Brain Behav Immun. 2007;21(6):727–35. Epub 2007/07/03. 10.1016/j.bbi.2007.05.005 . 17604598

[39] Besedovsky HO, del Rey A. Central and peripheral cytokines mediate immune-brain connectivity. Neurochem Res. 2011;36(1):1–6. Epub 2010/09/08. 10.1007/s11064-010-0252-x . 20820913

[40] Fenn AM, Henry CJ, Huang Y, Dugan A, Godbout JP. Lipopolysaccharide-induced interleukin (IL)-4 receptor-alpha expression and corresponding sensitivity to the M2 promoting effects of IL-4 are impaired in microglia of aged mice. Brain Behav Immun. 2012;26(5):766–77. Epub 2011/10/26. 10.1016/j.bbi.2011.10.003 22024136 PMC3288757

[41] Verma S, Nakaoke R, Dohgu S, Banks WA. Release of cytokines by brain endothelial cells: A polarized response to lipopolysaccharide. Brain Behav Immun. 2006;20(5):449–55. Epub 2005/11/29. 10.1016/j.bbi.2005.10.005 . 16309883

