# Supplementary figures and images for: Association between Prediagnostic Allergy-Related Serum Cytokines and Glioma
Source: PLoS One. 2015 Sep 9;10(9):e0137503. doi: 10.1371/journal.pone.0137503 (PMC4564184; doi:10.1371/journal.pone.0137503)

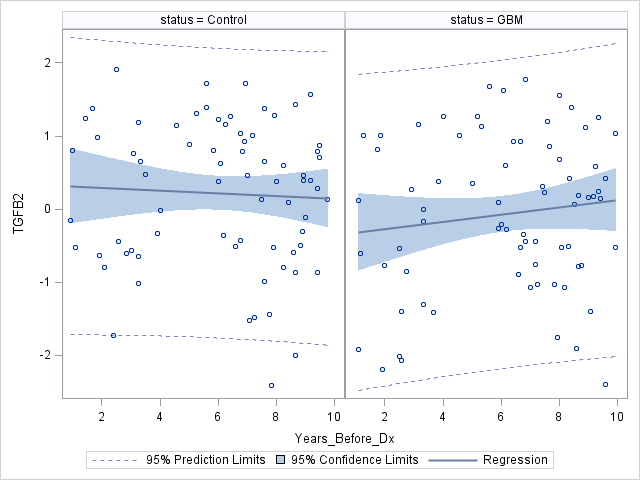

Supplement: S1 Fig — Graph is restricted to ten years before diagnosis. Graph on left shows the association among glioma controls (n = 72); graph on right shows the association among glioma cases (n = 73). (DOC) [file pone.0137503.s001.doc]

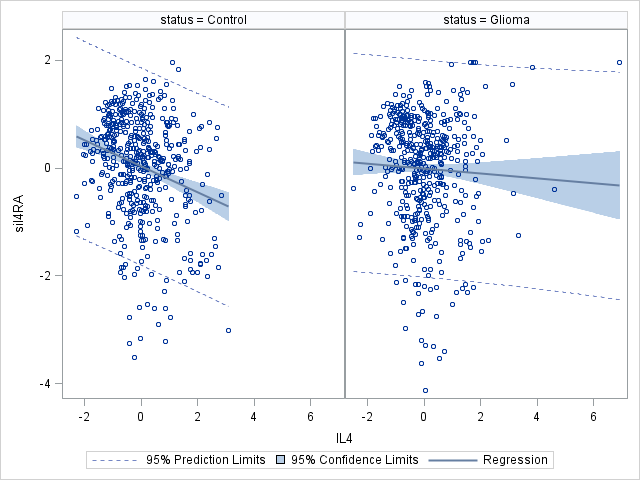

Supplement: S2 Fig — Graph on left shows the association among glioma controls (n = 487); graph on right shows the association among glioma cases (n = 487). (DOC) [file pone.0137503.s002.doc]

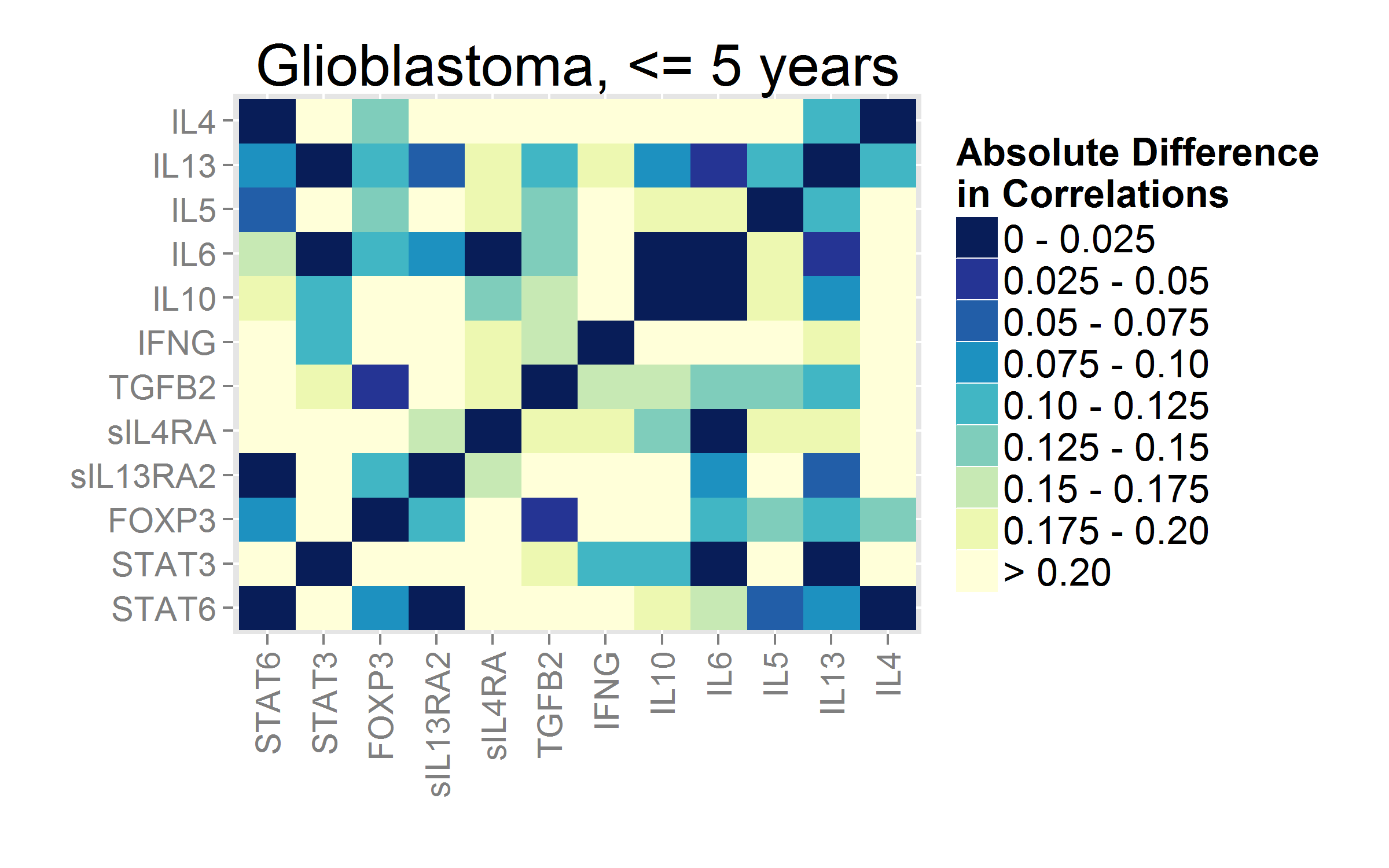


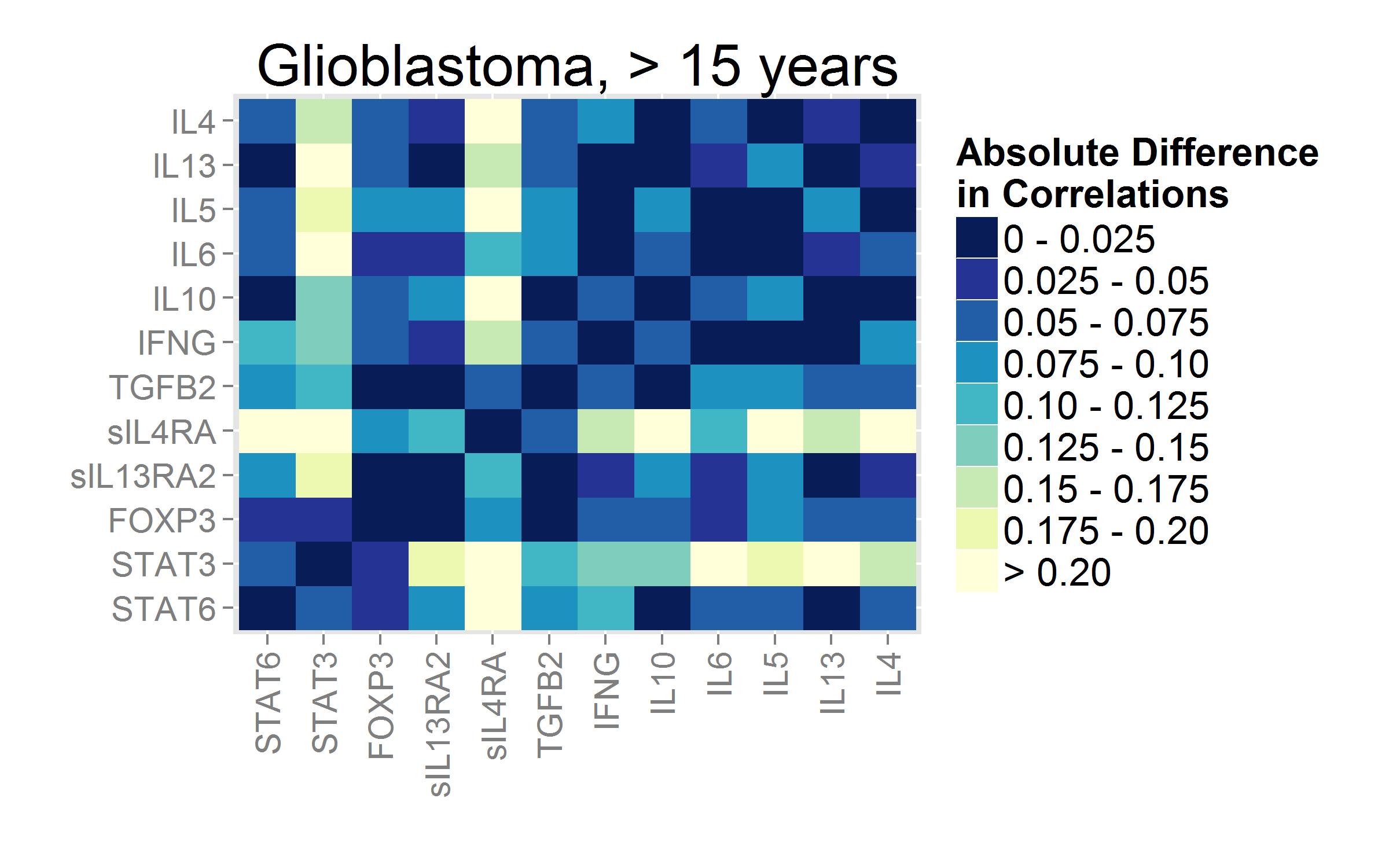

Supplement: S3 Fig — Lighter colors indicate larger absolute differences between case and control correlation coefficients. Top graph represents glioma cases and controls whose blood was drawn ≤ 5 years before diagnosis (n = 22 cases, 22 controls). Bottom graph represents glioma cases and controls whose blood was drawn > 15 years before diagnosis (n = 167 cases and 169 controls). (DOC) [file pone.0137503.s003.doc]
