# Supplementary material for: Association between Prediagnostic Allergy-Related Serum Cytokines and Glioma
Source: PLoS One. 2015 Sep 9;10(9):e0137503. doi: 10.1371/journal.pone.0137503 (PMC4564184; doi:10.1371/journal.pone.0137503)
Supplement: S1 Table — (DOC) [file pone.0137503.s004.doc]

**Supplemental Table 1. Median coefficients of variation (CV) by serum protein:**

**based on duplicate values collected in different batches.**

**Glioma Cases (n=47)**  **Controls (n=48)**

| **Serum Protein** | **Median** | **25th %** | **75th %** |
| --- | --- | --- | --- |
| | **IL4_CV** | | --- | | **IL13_CV** | | **IL5_CV** | | **IL6_CV** | | **IL10_CV** | | **IFNG_CV** | | **TGFB2_CV** | | **sIL4RA_CV** | | **sIL13RA2_CV** | | **FOXP3_CV** | | **STAT3_CV** | | **STAT6_CV** | | | 0.11 | | --- | | 0.02 | | 0.26 | | 0.02 | | 0.04 | | 0.02 | | 0.08 | | 0.10 | | 0.21 | | 0.06 | | 0.11 | | 0.05 | | | 0.06 | | --- | | 0.01 | | 0.16 | | 0.01 | | 0.02 | | 0.01 | | 0.05 | | 0.08 | | 0.16 | | 0.03 | | 0.02 | | 0.02 | | | 0.15 | | --- | | 0.03 | | 0.30 | | 0.02 | | 0.07 | | 0.04 | | 0.09 | | 0.11 | | 0.25 | | 0.08 | | 0.16 | | 0.07 | |

| **Serum Protein** | **Median** | **25th %** | **75th %** |
| --- | --- | --- | --- |
| | **IL4_CV** | | --- | | **IL13_CV** | | **IL5_CV** | | **IL6_CV** | | **IL10_CV** | | **IFNG_CV** | | **TGFB2_CV** | | **sIL4RA_CV** | | **sIL13RA2_CV** | | **FOXP3_CV** | | **STAT3_CV** | | **STAT6_CV** | | | 0.11 | | --- | | 0.02 | | 0.25 | | 0.02 | | 0.03 | | 0.02 | | 0.06 | | 0.09 | | 0.24 | | 0.06 | | 0.11 | | 0.06 | | | 0.07 | | --- | | 0.01 | | 0.18 | | 0.01 | | 0.02 | | 0.02 | | 0.05 | | 0.08 | | 0.17 | | 0.03 | | 0.06 | | 0.03 | | | 0.14 | | --- | | 0.03 | | 0.30 | | 0.03 | | 0.05 | | 0.04 | | 0.09 | | 0.11 | | 0.26 | | 0.10 | | 0.14 | | 0.09 | |
