# Supplementary material for: Association between Prediagnostic Allergy-Related Serum Cytokines and Glioma
Source: PLoS One. 2015 Sep 9;10(9):e0137503. doi: 10.1371/journal.pone.0137503 (PMC4564184; doi:10.1371/journal.pone.0137503)
Supplement: S2 Table — (DOC) [file pone.0137503.s005.doc]

| **Supplemental Table 2. Glioma case-control status by batch number** |
| --- |

**All glioma cases and controls**

| **Batch** | **1** | **2** | **3** | **4** | **5** | **6** | **7** | **8** | **9** | **10** | **11** | **6,12^1^** | **8,12^1^** | **Total** |
| --- | --- | --- | --- | --- | --- | --- | --- | --- | --- | --- | --- | --- | --- | --- |
| **Case** | 38 | 45 | 43 | 40 | 45 | 46 | 47 | 2^2^ | 42^3^ | 47 | 46 | 4 | 42 | 487 |
| **Control** | 37 | 38 | 42 | 46 | 43 | 43 | 45 | 2 | 49 | 46 | 48 | 4 | 44 | 487 |
|  |  |  |  |  |  |  |  |  |  |  |  |  |  |  |
| **Total** | 75 | 83 | 85 | 86 | 88 | 89 | 92 | 4 | 91 | 93 | 94 | 8 | 86 | 974 |
| **Glioma cases and controls ≤ 5 Years before diagnosis** | | | | | | | | | | | | | | |
| **Case** | 3 | 7 | 2 | 4 | 4 | 7 | 9 | --^4^ | 5 | 2 | 7 | --^4^ | 5 | 55 |
| **Control** | 3 | 5 | 3 | 2 | 5 | 6 | 11 | -- | 4 | 4 | 6 | -- | 6 | 55 |
|  |  |  |  |  |  |  |  |  |  |  |  |  |  |  |
| **Total** | 6 | 12 | 5 | 6 | 9 | 13 | 20 | 0 | 9 | 6 | 13 | 0 | 11 | 110 |

1. Samples measured in each batch, values averaged
2. One sample measured in batches 8, 9 and 12, values averaged
3. One sample measured twice , values averaged
4. No observations in batch
